# Temperatures leading to heat escape responses in Antarctic marine ectotherms match acute thermal limits

**DOI:** 10.3389/fphys.2022.1077376

**Published:** 2022-12-22

**Authors:** S. A. Morley, J. W. F. Chu, L. S. Peck, A. E. Bates

**Affiliations:** ^1^ British Antarctic Survey, Natural Environment Research Council, Cambridge, United Kingdom; ^2^ Pacific Enterprise Science Centre, Fisheries and Oceans Canada, West Vancouver, BC, Canada; ^3^ Biology Department, University of Victoria, Victoria, BC, Canada

**Keywords:** escape behaviour, thermal reaction norm, acute temperature, macrophysiology, polar marine

## Abstract

Thermal tolerance windows are key indicators of the range of temperatures tolerated by animals and therefore, a measure of resilience to climate change. In the ocean, where ectotherms are immersed, body temperatures are tightly coupled to environmental temperature and species have few options for thermoregulation. However, mobile species do have the ability to orientate towards optimal temperatures and move away from sub-optimal or dangerous temperatures. Escape responses are one such locomotory behavior, which typically manifests as a series of violent flicking movements that move individuals out of dangerous environments. We tested 11 species of Antarctic marine ectotherms, from one of the most stable shallow water marine environments, with an annual temperature range of −2°C to +2°C, that are vulnerable to small degrees of warming. Three species, the clam *Laternula elliptica*, the sea cucumber *Cucumaria georgiana*, and the brittlestar *Ophionotus victoriae*, showed no, or virtually no, escape response to temperature. Escape responses from a further eight species had a median response temperature of 11.2 (interquartile range, 10°C–15.7°C), which is well above current environmental temperatures but close to the range for acute lethal limits of Antarctic marine ectotherms (CT_max_ range, 17.2°C–26.6°C). This highlights that both acute tolerance limits and escape responses, fall outside current environmental temperatures, but also those predicted for 100s of years in the Southern Ocean. In a warmer Southern Ocean Antarctic fauna may not have the capacity to use temperature to select optimal thermal conditions, which leaves adaptation as a primary mechanism for their persistence.

## Introduction

Clear macrophysiological principles have emerged to describe how evolution of organism physiological capacities are shaped by the magnitude, variability and predictability of their experienced environment (Chown and Gaston, 2016). These relationships are used extensively to estimate the vulnerability of taxa at different latitudes to climate change (e.g., Gaston et al., 2009; Bennet et al., 2020). These principles are underpinned by the expectation that ectotherms have evolved to match an optimal environmental temperature range, within which their biochemical pathways, such as enzyme activity rates, work most efficiently (Hochachka and Somero 2002). Their importance in determining species distributions is evidenced by species migrating polewards and to higher latitudes to track climate warming and remain within their optimal thermal envelopes (Burrows et al., 2014). Central to this study is the concept that the breadth of thermal reaction norms match the variability and predictability of the environment that organisms experience (Gaston et al., 2009).

**FIGURE 1:**
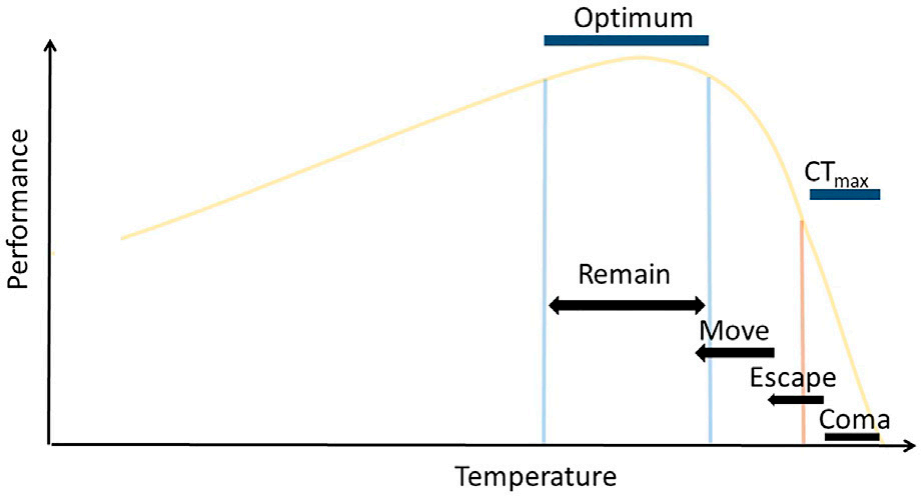
Theoretical thermal reaction norm for performance of a trait, highlighting the expected locomotory response of mobile marine ectotherms to increasing thermal exposure. Exposure to extreme temperatures above a threshold (orange line), for sufficient duration, will result in animals entering heat coma at CT_max_, losing the ability to escape lethal temperatures. Short duration exposure to temperatures above this threshold, or extended exposure to temperatures close to this threshold, are expected to illicit escape responses that will move individuals to cooler temperatures. Individuals exposed to temperatures above their optimum range are expected to also move to cooler temperatures and relocate within their optimal temperature range, where they will remain.

Sessile ectotherms have limited opportunities to thermoregulate, but mobile ectotherms can use behavioural responses to avoid extreme temperatures and remain in suitable habitat. Locomotion that orients individuals within optimal conditions have been relatively well-studied (Figure 1; the “final preferendum hypothesis,” Reynolds and Casterlin, 1979). Such behaviours are common in ectotherms, particularly reptiles, insects and fishes to achieve the regulation of daily body temperatures and are closely correlated with their acute thermal limits (e.g., Bates et al., 2010; Sunday et al., 2014). Temperatures on either side of the optimal range lead to sub-optimal body temperatures and organism performance reduces (e.g., Pörtner 2002). The breadth and shape of, what are called, thermal reaction norms, have become key tools for describing species vulnerability to environmental variability (Huey and Kingsolver, 1989) and for testing for common underlying mechanisms. Several paradigms have been developed from these relationships that allow comparisons of the relative vulnerabilities of species across latitudes (e.g., Addo-Bediako et al., 2000).

A key question given extreme heat events which are increasing in magnitude and frequency in the ocean, is, what mechanisms lead to survival and persistence? Different physiologies have different thermal limits, and functions critical for survival, such a righting, can have a higher thermal limit than less time critical functions such as feeding (Pörtner, 2002; de Leij et al., 2022). Thermal limits can also vary with the rate at which they are warmed (de Leij, et al., 2002). At extreme temperatures, approaching those that are high enough to result in heat coma, escape response behaviours may be a last resort mechanism. Escape responses are typically a series of rapidly expressed violent swimming or flicking movements that are atypical, and lead to individuals rapidly moving out of dangerous environments. Such behaviours are more commonly studied in relation to predator avoidance, but such behaviours have also evolved as a mechanism to escape extreme heat (e.g., Bates et al., 2010; Patterson-Kane et al., 2018).

Our study focused on marine ectotherms from the Southern Ocean, which has one of the most stable, cold, near shore, annual temperature ranges, of less than 4°C (−2°C to +2°C). Polar marine ectotherms are characterised as being highly vulnerable, with a low capacity to survive acute warming (Peck et al., 2009), coupled with generally poor acclimation capacities and long generation times that reduce opportunities for adaptation (Peck et al., 2014). While some of the physiological mechanisms underlying the vulnerability of Antarctic marine ectotherms are well understood (Pörtner, 2002; Peck et al., 2009; Peck et al., 2014; de Leij et al., 2022), others such as behavioural responses to environmental cues have been less well studied. A previous study by Bates et al. (2010) indicated that movement in a thermal gradient was limited and suggested that this poor thermal behavioural response may be a result of their evolutionary environment in the Antarctic. We test this hypothesis through experiments to see if marine invertebrate species from these cold, stable conditions express an escape response to acute thermal challenge, and if they do respond, whether this response is triggered at ecologically relevant temperatures.

## Materials and methods

In order to minimize the size range of individuals between treatments, average size adults of 11 species of Antarctic marine ectotherms were collected by SCUBA divers at depths between 6 and 20 m from bays surrounding Rothera research station, Antarctica (67.57°S, 68.12°W; Table 1). Individuals were given at least 24 h to recover from collection, in a flow-through aquarium, at ambient temperature of 1.3°C ± 0.2, before experiments were initiated.

**TABLE 1:** Sample size of each species at each temperature.

Species	Nominal temperature, °C	Number of individuals	Number of escape responses	Number in heat coma
*Antarctonemertes* spp.	1	5	0	0
	5	5	0	0
	10	5	3	0
	15	5	5	0
*Parborlasia corrugatus*	1	5	0	0
	8	4	0	0
	10	5	5	0
	15	5	5	0
*Ophionotus victoriae*	1	10	0	0
	5	10	0	0
	8	6	0	0
	10	10	0	0
	15	2	2	0
*Cucumaria georgiana*	1	10	0	0
	5	10	0	0
	10	8	0	0
	15	10	0	0
*Aeguiyoldia eightsii*	1	10	0	0
	5	10	0	0
	10	10	3	0
	15	10	3	0
	20	5	1	0
*Laternula elliptica*	1	5	0	0
	1	5	0	0
	10	5	0	0
	15	5	0	0
Amphipod_B	1	10	0	0
	5	5	0	0
	10	10	2	0
	15	10	10	0
*Barrukia* spp.	1	15	0	0
	5	15	2	0
	8	4	2	0
	10	15	7	0
	15	15	13	1
*Paraceradocus miersi*	1	15	0	0
	5	15	4	0
	8	5	4	0
	10	15	10	0
	15	16	10	6
*Prostebbingia* sp.	1	5	0	0
	5	5	0	0
	10	5	3	2
	15	5	4	0
*Munna antarctica*	1	10	0	0
	5	10	6	0
	10	10	8	0
	15	5	—	5

To maintain constant temperatures during each trial, three beakers of seawater were placed in a temperature controlled jacketed water bath controlled by a Grant Instruments LTD50G heating/cooling thermocirculator. Room temperature (20°C) and aquarium temperature (1.3°C) water were mixed to achieve the required treatment temperatures, 1.3°C ± 0.6 (mean ± SD), 5.1°C ± 0.2, 9.9°C ± 1.4, 15.8°C ± 0.4, and 20.1°C ± 0.3. Water was discarded after every trial and the beakers were washed and dried before fresh seawater was mixed to the next treatment temperature. The beakers were cleaned and filled with new seawater to ensure that there was no influence of any chemical release by individuals from one trial impacting on behavior of animals in the subsequent trial.

A LifeCam Studio HD camera (Microsoft) was suspended directly above the beakers and behaviour was recorded using Video Velocity software (Candy Labs.) onto a computer hard drive. The time-lapse video (five frames per second) was set to record before one animal was introduced into each 2 L beaker. Individuals were captured separately from the tank and gently released at the water surface of each of the beakers. The response of animals at 1.3°C were classified as control behaviours (Table 2). This was to ensure that any response due to being handled was documented allowing additional behaviours, beyond these control responses, to be recorded using an ethogram approach. Behaviours classified as an acute escape response were those that would act to rapidly move the animal away from, in this case, water of harmful temperature. These were catalogued as “violent” or sudden, changes in locomotion, speed, velocity, acceleration, orientation, bending of the body, turning or other specific activities, such as foot probing attempts in bivalves, beyond those recorded in control animals (Table 1).

**TABLE 2:** Control and escape behaviours of 11 species of Antarctic marine invertebrates.

Species	Control behavior	Escape response
*Antarctonemertes* spp.	Uncoiling and then moving	Head lifting and snaking. Rolling over and over
*P. corrugatus*	Extending followed by slow left and right head movements	Multiple extensions and contractions. Head snaking. Writhing and coiling
*O. victoriae*	Moving to the edge of beaker, with constant arm movements	Rapid arm movement
*C. georgiana*	Slowly extending tentacles	None
*A. eightsii*	No response or shell gaping and siphon extension	Foot probing, moving shell
*L. elliptica*	Siphons slowly extending	None
*Barrukia* spp.	Turning over, stoping, starting to crawl	S-swimming; swimming by curving the body into an S-shape
Amphipod_B	Swimming and sinking. Continuous swimming around edge of beaker	Tail flicking with no swimming
*P. miersi*	1–3 initial tail flicks, walking around edge of beaker with occasional turns	Continuous tail flicking (>5), sometimes followed by walking with multiple turns
*Prostebbingia* sp.	Swimming and sinking. Continuous swimming around edge of beaker	Continuous tail thrashing with lack of directional swimming
*M. antarctica*	Some walking but generally still	Thrashing of legs and bucking of body

As escape responses are, by their very nature, very rapid, almost instantaneaous, reactions to negative stressors, recording was stopped after 5 min, or earlier if a previously active animal went into heat coma for more than 1 min. Heat coma was defined as an absence of any movement and was confirmed by a lack of response of appendages, tentacles or body tissues to a stimulus with a blunt seaker (*cf* Peck et al., 2009). This is typically referred to as CT_max_, the upper temperature at which an animal suffers a loss of equilibrium. Behaviours were visually documented during each trial but also confirmed afterwards through video review. 4 to 16 individuals were used in each temperature trial for each species [Mean 8.4 ± 0.6 (±SE Table 1)]. This is with the exception of one trial of *O. victoriae* where the two remaining individuals were tested at a higher temperature of 15°C. All individuals were returned back to the flow through aquarium and checked after 24 h to ensure they had recovered to normal locomotory and behavioural response levels.

Escape response temperatures were not normally distributed and so Kruskal-Wallis tests were completed followed by Dunn’s multiple comparison tests with the probability of acceptance adjusted accordingly (R-packages: dplyr, Wickham et al., 2022; FSA, Ogle et al., 2022). Median escape response temperature from this study was compared with previously published median upper temperature limits (CT_max_) for eight of the 11 species (at a heating rate of 1°C day^−1^; Peck et al., 2009). The relationship was tested with a regression analysis (Minitab 19).

## Results

**FIGURE 2:**
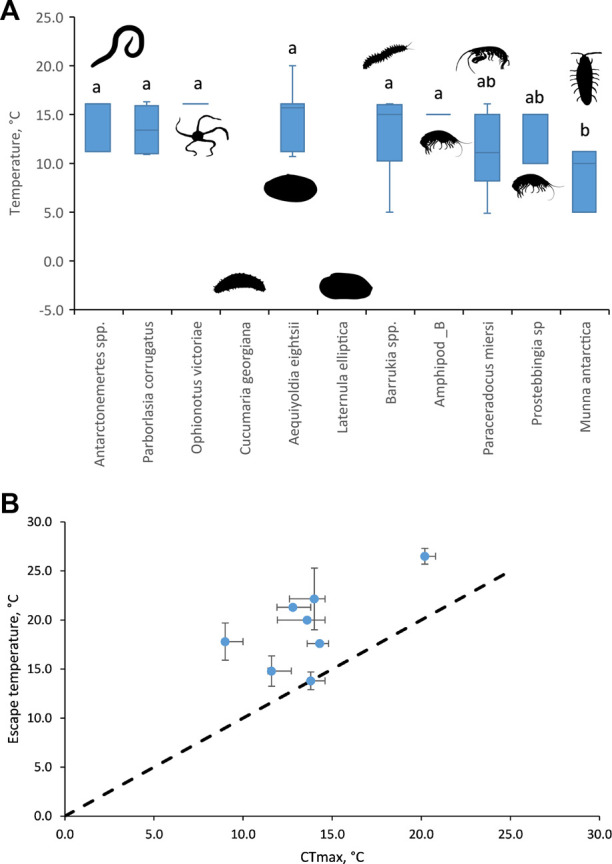
**(A)** Temperatures at which escape responses were recorded for each of the 11 species tested. Boxes are median values with upper and lower interquartile ranges. The lack of a box plot indicates that no escape response was recorded for *C. georgiana* and *L. ellipitca*. *O. victoriae* and Amphipod_B only responded at one temperature. Silhouettes represent the taxon of each species. Species with the same lowercase letters indicate that escape responses were not significantly different, Dunn’s non-parametric post-hoc test. **(B)** Escape temperatures plotted against CT_max_ (at a warming rate of 1°C h^−1^, data available for eight of the 11 species in this study from Peck et al., 2009). Median ± interquartile range (missing interquartile ranges indicate small sample sizes, or responses at a single temperature, as detailed above).

Three of the 11 Antarctic species either showed no escape response at any temperature (the clam *L. elliptica* and the sea cucumber *Cucumaria georgiana* did not move within the 5 min) or virtually no escape response (only two individuals of the brittlestar *O. victoriae* responded; Table 2; Figure 2A). In eight species, escape responses ranged from foot probing (e.g., *Aequiyoldia eightsi*) to strong thrashing movements of the body (e.g., *Munna antarctica*; Table 2). Escape behaviours were triggered at temperatures ranging from 5 to 20°C (median 11.2, interquartile range, 10°C–15.7°C; Figure 2A) and were consistent responses within species. There was a significant difference in the temperature of escape response between species (Kruskal-Wallis, Chi^2^ = 29.29, *p* < 0.01). The isopod, *M. antarctica*, had a significantly lower median escape response temperature (Dunn’s test; Z > 3.1, adj.*p* > 0.05) to all species except the amphipods, *Paraceradocus meirsi* (Z = 2.2, adj.*p* = 0.11) and *Prostabbingia* sp. (Z = 1.8, adj.*p* = 0.20).

Previously published lethal limits, at a warming rate of 1°C h^−1^, (Peck et al., 2009) were available for eight of the 11 species tested for escape responses. Published lethal limits were not available for *Antarctonemertes* spp., Amphipod_B, and *Prostebbingia* sp. All but one of the lethal limits (Peck et al., 2009) were above the escape responses (above the dotted line in Figure 2B). Only *M. antarctica* had equal median lethal and escape response temperatures (13.8°C, Figure 2B). There was no significant linear relationship between the medians of the two temperarture limits [F_(1,7)_ = 4.68, *p* = 0.07].

## Discussion

Three out of eleven Antarctic marine ectotherms tested here either did not exhibit a heat escape response, or, in the case of *O. victoriae*, had a limited response, with only two individuals responding within 5 min within our temperature range. The weak escape responses measured in the current study corresponds with observations during a behavioural temperature preference study of Antarctic marine invertebrates in a temperature gradient, when only two of the 12 Antarctic species tested by Bates et al. (2010) displayed an escape response on exposure to warm temperatures. During these thermal gradient experiments, some individuals remained at temperatures around 7°C that were warm enough to elicit heat coma and would ultimately lead to death [Cumaceans (*Eudorella splendida*), errant polychaetes (*Nephtydae*) and nemertean worms (*Parborlasia corrugatus*); Bates et al., 2010]. *L. elliptica* does exhibit escape responses when removed from the sediment (Ansell and Rhodes, 1997), exhibiting burrowing, looping and water jetting. These behaviours were not, however, triggered by temperature in the current experiment. Escape response behaviours are typically observed as an instantaneous response to the negative stimulus, but as behavioural responses are often slower in Antarctic marine ectotherms (Peck et al., 2004). It is possible that additional responses may have been measured if experiments had been run for longer duration, although, it is more likely that these temperatures will illicit heat coma than a delayed escape response. This lack of strong heat escape responses in Antarctic marine invertebrates may not be surprising, given that the Southern Ocean has experienced cold stable temperatures for more than 15 *mya* (Crame, 1993). In contrast, hydrothermal vent and intertidal species displayed marked and strong heat escape responses across diverse species (Bates et al., 2010). Heat escape responses are presumably required in habitats where the probability of being exposed to temperature extremes is high.

In behavioural choice experiments in a thermal gradient, many individuals also did not move away from harmful temperatures. The selected temperature range of Antarctic species, that did move (0.1°C–3.4°C; Bates et al., 2010), was much closer to the environmental temperature range in the Southern Ocean (−1.9°C to 2.0°C; Morley et al., 2022). This range also corresponds to the estimated long-term temperature limits for species in this assemblage (1°C–6°C over 2–5–months, Peck et al., 2009). Upper temperature limits in Antarctic marine ecototherms vary between physiological processes and with the rate of warming (de Leij et al., 2022). The species and individuals exhibiting an escape response, in the current study, did not respond until a median temperature of 11.2°C (10°C–15.7°C). This range is above environmental temperatures but was largely below the CTmax measured for eight of these species warmed at an acute rate of 1°day^−1^ (17.2°C to 26.6°C, Peck et al., 2009 with additional unpublished data). It is tempting to suggest that escape response thresholds in Antarctic marine ectotherms have evolved in line with their acute limits rather than in response to current day ecological thresholds in the cold stable Southern Ocean.

It is not known whether acute escape responses and acute thermal limits are mechanistically linked. In fact there is growing evidence that multiple mechanisms underlie thermal limits for different physiological processes (Lefevre et al., 2021; de Leij et al., 2022; Jorgensen et al., 2022). Behavioural and biochemical protective responses to temperature rely on an individual’s ability to sense and respond to suboptimal temperature exposure. Such behavioural capacity seems an obvious adaptation for variable thermal environments, such as hydrothermal vents or intertidal locations (Bates et al., 2010), but has not previously been recorded in species from the subtidal Antarctic where thermal variation is minimal. For these acute behavioural responses to be triggered by temperature, cells need to have a biochemical mechanism for detecting when ambient temperature becomes sub-optimal. The sub-optimal temperature rage at which *L. elliptica* starts to produce anaerobic end products, one of the indicators of sub-optimal temperatures (Pörtner et al., 2002), is between 3°C and 10°C (Morley et al., 2009; Truebano et al., 2010), temperatures that animals were exposed to in this experiment. However, the acute exposure times in this experiment may have been too short to elicit such biochemical triggers. Various molecular strategies that enable cells to detect absolute, as well as the change, in temperature have been identified (Sengupta and Garrity, 2013). Most trigger mechanisms involve conformational changes in secondary or tertiary molecular structures that alter their function at temperature thresholds, e.g., the unfolding of mRNA that allows transcription to take place. Our results suggest that selective pressure for the evolution, or retention, of mechanisms to respond are weak for heat escape responses, as would be predicted under macrophysiological expectations in such a stable temperature regime. Indeed, the range of temperatures that elicit an escape response may simply be a remnant from evolutionary history (Bennet et al., 2021), alternately, the evolution of acute thermal escape responses could be mechanistically linked to both the magnitude and variability of extreme, rather than climatic temperatures.

This study highlights the importance of behaviour as an adaption to cope with thermal stress, and thus a trait to consider in species climate change vulnerability assessments. In a warming world we need to determine the drivers of key traits that promote resilience and over what time scale this resilience will prove to be important. Our findings also highlight that acute tolerance limits are coupled with heat escape responses that fall far outside what is typical for Antarctica now, and is much higher than ocean temperatures predicted for 100s of years. Antarctic fauna may have lost or only have weak capacity to detect ecologically relevant acute high temperatures. If they exhibit a similar response to longer duration exposures, then this will also reduce their ability to select optimal thermal conditions. Combined with their reduced adaptive capacity, this raises further concerns over the persistence of Antarctic fauna.

## Data Availability

The original contributions presented in the study are included in the article/Supplementary Material, further inquiries can be directed to the corresponding author.
